# Association of frailty and malnutrition with pneumonia severity in PCV-13 vaccinated older adults: a single-centre experience

**DOI:** 10.1186/s12877-025-06736-5

**Published:** 2025-12-17

**Authors:** Yasemin Polat Özer, Zeynep Şahiner, Merve Güner, Kerim Çayıröz, Emir Lütfü Kılıç, Cafer Balcı, Mertcan Uzun, Burcu Balam Doğu, Mustafa Cankurtaran, Ahmet Çağkan İnkaya, Serhat Ünal, Meltem Gülhan Halil

**Affiliations:** 1 Division of Geriatrics, Department of Internal Medicine, Hacettepe University Faculty of Medicine, Ankara, Turkey; 2 Department of Internal Medicine, Hacettepe University Faculty of Medicine, Ankara, Turkey; 3 Department of Infectious Diseases and Clinical Microbiology, Hacettepe University Faculty of Medicine, Ankara, Turkey

**Keywords:** Immunisations, Frailty, Malnutrition, Older adults, Pneumococcal conjugate vaccine

## Abstract

**Background:**

Pneumonia remains a leading cause of morbidity and mortality in older adults, particularly due to the age-related decline in immune function. The aim of this study was to evaluate the association between frailty, malnutrition, and the severity of pneumonia in older adults who received the 13-valent pneumococcal conjugate vaccine (PCV13).

**Methods:**

A retrospective analysis was made of 407 geriatric outpatients aged over 65 years who had received PCV13. Clinical and radiological data were collected from electronic health records and verified via telephone interviews. The primary outcomes were incidence and severity of pneumonia (mild vs. severe), hospitalization, mortality, and antibiotic use within one year following vaccination. Pneumonia severity was classified according to established criteria in the literature.

**Results:**

Of the 407 patients evaluated (mean age 73.3 ± 6.3 years; range 65–91 years; 62.9% female), 50 (12.2%) developed pneumonia within one year after PCV13 vaccination. Pneumonia was more common in patients with diabetes mellitus (*p* = 0.003) and chronic obstructive pulmonary disease (*p* < 0.001). Severe pneumonia was significantly more prevalent among frail individuals (*p* = 0.006). Compared to patients with mild pneumonia, those with severe pneumonia had higher Clinical Frailty Scale scores and prolonged chair stand test durations (*p* = 0.002 and *p* = 0.031, respectively). After adjusting for age, sex, and frailty, malnutrition emerged as an independent risk factor for severe pneumonia (OR: 11.9, 95% CI: 1.592–89.285, *p* = 0.016).

**Conclusion:**

The findings of this study indicate that malnutrition and frailty are independent risk factors for severe pneumonia diagnosed within a year of PCV13 vaccination in older adults. Consequently, comprehensive geriatric assessment and targeted interventions beyond vaccination are essential to reduce pneumonia risk in frail or malnourished older populations.

**Trial registration:**

Not applicable.

## Introduction

The bacterium Streptococcus pneumoniae (S. pneumoniae) is responsible for 27% of pneumonia diagnoses worldwide, causing 250,000 hospital admissions and 18,000 deaths among older adults annually [1–3]. Furthermore, the treatment of pneumococcal disease results in an annual expenditure of 3.5 billion US dollars, the majority of which is attributable to the management of older adults [4, 5]. The heightened pneumococcal disease risk in older adults is predominantly due to age-related immune system changes, known as immunosenescence. The prevention of pneumococcal disease in the older population is contingent on conjugate or polysaccharide pneumococcal vaccines.

The 13-valent Pneumococcal Conjugate Vaccine (PCV13) is effective against 13 serotypes of S. pneumoniae. In addition to eliciting T-cell responses, it has been shown to induce the secretion of IgG-type antibodies from B lymphocytes and the development of memory B cells [6]. Pneumococcal conjugate vaccines are effective in preventing invasive disease in older adults, although their effectiveness is known to decrease with age. The vaccine is 90% effective in children, decreasing to 72.8% in older adults [7, 8].

PCV13 and the 23-valent Pneumococcal Polysaccharide Vaccine (PPSV23) are routinely used in adults in most countries, including Turkey, to prevent pneumococcal diseases in people aged over 65 years [9]. In 2014, the recommendation was made for PCV13 to be administered in conjunction with PPSV23 for all adults aged ≥ 65 years [10–13]. In addition to its capacity to reduce mortality and morbidity, pneumococcal vaccination has been reported to decrease antimicrobial consumption, thereby contributing to a reduction in antimicrobial resistance (AMR) [14, 15].

Malnutrition is known to be one of the most significant geriatric syndromes. It is a well-documented fact that malnutrition is associated with a range of adverse clinical outcomes, including various morbidities and mortality [16]. Another prevalent geriatric syndrome is frailty, which occurs when physical and psychological stress factors diminish physiological reserve. Frailty is associated with adverse outcomes such as disability, hospitalization, and nursing home admission [17]. Moreover, malnutrition exerts a pivotal role in the pathophysiology of sarcopenia and frailty, which are intricately linked.

The hypothesis of this study was that PCV-13 vaccination alone may not fully mitigate pneumonia severity in vulnerable individuals, as frailty and malnutrition could impair immune response, reduce physiological reserve, and increase susceptibility to severe infections and poor outcomes. The aim of the study was to evaluate the impact of frailty and malnutrition on pneumonia severity in PCV-13-vaccinated older adults.

## Material and method

### Study design and population

This retrospective, single-centre analysis recruited 407 participants aged over 65 years, who presented at the Geriatric Medicine Clinics of Hacettepe University Faculty of Medicine Adult Hospital between December 2021 and December 2022. All participants had previously received a vaccination for PCV-13 and had not received PPSV23. The study inclusion criteria were defined as voluntary participation, and not meeting any of the exclusion criteria. The exclusion criteria for patients were stated as follows: active infection, newly diagnosed or non-complete remission malignancy, hospitalization or surgery within the last month, and lack of contact with the interviewers. A total of 22 patients were excluded due to missing data, and 12 because they could not be reached by telephone. Ultimately, 407 patients with complete data and who could be contacted by telephone were included in the study.

The data were collected from hospital records during the study period. The rates of hospitalization and mortality, pneumonia, and antibiotic use within one year after pneumococcal vaccination were obtained from National Health System records through the hospital automation program, and e-Nabız^®^, a nationwide electronic health records service provided by the Turkish Ministry of Health. The patients were called by telephone, and their pneumonia status was reconfirmed.

### Patient characteristics

The demographic data of the participants, including age, sex, education, and marital status, as well as information on chronic diseases, medications, and laboratory values (electrolytes including sodium, potassium, calcium, phosphorus, sedimentation rate, C reactive protein, serum albumin, 25-OH-vitamin D, thyroid stimulating hormone, and complete blood count), were recorded from patients’ files, retrospectively.

A comprehensive geriatric assessment (CGA) was performed using standardised tools and then recorded in the patient files. The Katz Index of Independence in Activities of Daily Living (ADL; score 0–6) and the Lawton–Brody Instrumental ADL (score 0–8) were utilised to evaluate functionality [18, 19]. The Standardized Mini-Mental State Examination (SMMSE) was used to assess orientation, memory, attention, calculation, recall, language, motor function, and perception skills [20]. Nutritional screening was conducted using the Mini-Nutritional Assessment short-form (MNA-SF). Scores of ≥ 11 points were classified as normal, 8–11 points as at risk of malnutrition, and scores of ≤ 7 as being malnourished [21]. The Yesavage Geriatric Depression Scale (YGDS) was used to screen for depression, with patients scoring ≥ 5 points being assessed clinically for depression [22]. The Clinical Frailty Scale (CFS) was used to evaluate frailty, and patients were diagnosed as living with frailty when the score was ≥ 4 [23].

Muscle strength was measured using a hand-grip dynamometer (Grip Strength Takei dynamometer, Niigata City, Japan). The participants were instructed to exert maximum force while performing this task. Three attempts were made, with a one-minute interval between each attempt. The measurement with the highest value was then selected for further analysis. The unit of measurement employed for the results was kilograms. The thresholds of 16 kg for women and 27 kg for men were accepted, in accordance with the recommendations outlined in the EWGSOP-2 [24]. Walking speed was measured using a 4-m course. Slow walking speed was defined as ≤ 0.8 m per second [24]. The chair-stand test (CST) and timed-up-and-go test (TUG) were conducted to measure muscle strength and physical performance. All the performance tests were performed twice, and the average of the two measurements was recorded. A cut-off value of ≥ 15 s was used for the CST and ≥ 20 s for the TUG test [24]. CGA evaluations performed within three months before and after vaccination were recorded. All the patients included were those who had attended our outpatient clinic and had received the PCV-13 vaccine, as documented in the hospital records. The PCV-13 vaccine is routinely recommended for adults aged ≥ 65 years as part of immunization practices within the scope of CGA.

### Assessment of pneumonia and its severity

**Fig. 1:**
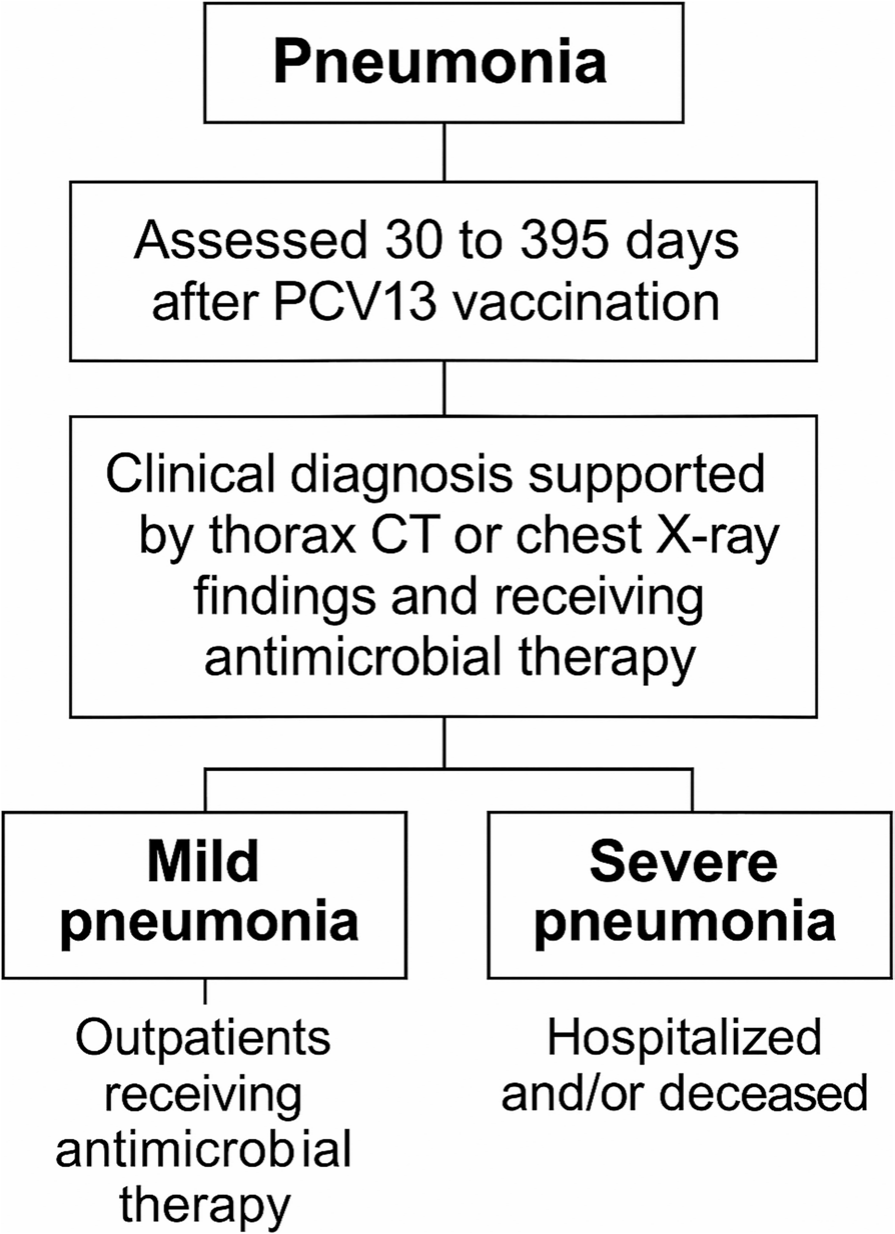
Assessment of pneumonia and its severity

Pneumonia was retrospectively defined based on clinical, radiological, and antimicrobial treatment criteria. Clinical data were obtained from epicrisis reports and patient self-reports via telephone calls, supported by thorax computed tomography (CT) or chest X-ray findings within 30 to 395 days after PCV13 vaccination. All patients diagnosed with pneumonia had available radiological imaging (thorax CT or chest X-ray). Antimicrobial treatment was used as a definitive criterion for both diagnosis and severity assessment. Data on the ambulatory status, hospitalization, intensive care unit admission, and survival of the patients were also recorded. Patients who received antimicrobial therapy as outpatients were classified as having mild pneumonia, and those who were hospitalized and/or died were categorized as having severe pneumonia, as shown in Fig. 1.

### Statistical analysis

Data obtained in the study were analyzd statistically using Statistical Package for Social Sciences (SPSS) for Windows vn. 22 software (IBM SPSS Inc., Chicago, IL, USA). The conformity of variables to normal distribution was examined using visual (histogram and probability graphs) and analytical methods (Shapiro-Wilk test and Kolmogorov-Smirnov test). Descriptive statistics were stated as mean ± standard deviation (SD) values for normally distributed variables, and median [maximum-minimum] values for non-normally distributed variables. Categorical variables were expressed as a number (n) and percentage (%). The Chi-square and Fisher’s Exact Test were used to compare categorical variables in the two groups. In the comparison of the two groups, normally distributed continuous variables were analyzed with an independent t-test, and non-normally distributed continuous variables with the Mann-Whitney U-test. Binary logistic regression analysis with backward selection was performed within the two groups and adjusted for confounding variables according to the univariate analysis. A value of *p* < 0.05 was accepted as the level of statistical significance.

## Results

Evaluations were made of a total of 407 patients, comprising 256 (62.9%) females and 151 (37.1%) males with a mean age of 73.3 ± 6.3 years (range, 65–91 years). From the entire study group, those who had pneumonia within 1 year after PCV-13 vaccination (*n* = 50, 12.2%) were categorised as the pneumonia group, and those who did not have pneumonia (*n* = 357, 87.8%) were considered as without pneumonia. The comparisons of demographic, clinical, and CGA parameters between patients with and without pneumonia after PCV-13 are summarised in Table 1.

**Table 1:** Comparisons of demographic, clinical, and comprehensive geriatric assessment characteristics between patients with and without pneumonia

	Pneumonia group (*n* = 50)	Without pneumonia (*n* = 357)	*p*
Age, years	73.4 ± 6.7	72.4 ± 5.9	0.25
Sex, female	31(62.0)	225(63.0)	0.888
BMI kg/m ^2^	28.2 ± 7.1	29.4 ± 7.2	0.281
BMI (≥ 30)	16(34.8)	149(44.5)	0.213
Education (> 8 years)	9(19.1)	91(26.3)	0.489
Smoking history	14(30.4)	97(28.2)	0.752
Marital status, married	32(68.1)	205(59.2)	0.245
Chronic conditions			
COPD	16(32.0)	29(8.2)	**< 0.001**
Hypertension	38(76.0)	260(73.0)	0.657
Coronary artery disease	14(28.0)	100(28.2)	0.980
Diabetes mellitus	31(62)	143(40.1)	**0.003**
Malignancy	7(14.0)	45(12.6)	0.788
Chronic kidney disease	7(14.0)	25(7.1)	0.096
Multimorbidity (≥ 2 chronic diseases)	40(81.6)	277(79.4)	0.713
Polypharmacy (≥ 5 drugs)	27(56.3)	183(52.0)	0.579
Comprehensive geriatric assessment			
Katz ADL	6.0[0.0]	6.0[0.0]	0.61
Lawton-Brody IADL	8.0[2.0]	8.0[1.0]	0.60
Clinical frailty scale score	4.0[1.0]	3.0[1.0]	0.76
MNA-SF score	13[2.0]	14.0[2.0]	0.78
Living with frailty (CFS ≥ 4)	26(55.3)	191(55.5)	0.98
Malnutrition (MNA-SF ≤ 11)	11(23.4)	96(28.1)	0.50
Probable sarcopenia	21(53.8)	129(45.1)	0.30
Handgrip strength, kg	19 [11.6]	21.0 [11.1]	0.44
4 m walking, sec	4.4 ± 2.1	4.4 ± 2.0	0.90
TUG test, sec	12.7 ± 6.6	11.4 ± 4.4	0.30
Chair stand test, sec	16.0 ± 9.3	15.2 ± 5.2	0.19
YGDS	2.0 [4.0]	2.0 [4.0]	0.82
SMMSE	27.0 [4.0]	28.0 [5.0]	0.67
Laboratory parameters			
Fasting blood glucose, mg/dL	111.0 ± 32.0	104.0 ± 30.0	0.071
Creatinine, mg/dL	0.8 ± 0.3	0.8 ± 0.4	0.241
Magnesium, mg/dL	1.9 ± 0.5	2.0 ± 0.3	0.155
Leukocyte, ×10³/µL	6.3 ± 2.4	6.9 ± 2.7	0.846
Neutrophil, ×10³/µL	3.7 ± 1.8	4.1 ± 1.9	0.883
Lymphocyte, ×10³/µL	1.8 ± 1.0	1.9 ± 0.9	0.373
HbA1c, %	6.1 ± 1.2	6.2 ± 1.3	0.155
25-OH-Vitamin D, ng/mL	21.0 ± 16.0	20.0 ± 16.0	0.815
C-reactive protein, mg/L	0.3 ± 1.0	0.4 ± 0.6	0.672
Sedimentation rate, mm/h	12.0 ± 12.0	14.0 ± 17.0	0.434

Variables are presented as n (%), mean ± SD, median [IQR] values and bold values indicate statistical significance (*p* < 0.05)

*ADL* Activities of daily living, *COPD* Chronic obstructive pulmonary disease, *MNA-SF* Mini-nutritional assessment short-form, *SMMSE* Standardized mini-mental state examination, *TUG* Timed-up-and-go, *YGDS* The yesavage geriatric depression scale

The mean age of patients was 73.4 ± 6.7 years in the pneumonia group and 72.4 ± 5.9 years in the group without pneumonia (*p* = 0.25). The presence of chronic conditions was examined and diabetes mellitus (DM) and chronic obstructive pulmonary disease (COPD) were found to be statistically more prevalent in the pneumonia group than in the non-pneumonia group (*p* = 0.003, *p* < 0.001, respectively). The detailed results are shown in Table 1. No significant differences were observed between the pneumonia and non-pneumonia groups in respect of the CGA parameters (Table 1).

The patients with pneumonia were divided into two subgroups: those with pneumonia as an outpatient with the use of antibiotics were included in the mild pneumonia group (*n* = 36, 72.0%), and those who were hospitalised on a ward or in the intensive care unit and/or died of pneumonia were included in the severe pneumonia group (*n* = 14, 28.0%). The comparisons of demographic, clinical, and CGA characteristics between the patients with mild and severe pneumonia are summarised in Table 2. Patients with severe pneumonia were determined to be significantly older than those with mild pneumonia (mean age: 74.8 ± 5.9 vs. 71.4 ± 6.0 years, *p* = 0.041). The CFS score and CST time were higher in the severe pneumonia group than in the mild pneumonia group (*p* = 0.002 and *p* = 0.031, respectively). The proportion of patients living with frailty was significantly higher among those with severe pneumonia compared to those with mild pneumonia (85.7% vs. 42.4%, *p* = 0.006). The median MNA-SF score was lower in those with severe pneumonia, and malnutrition was significantly more common (*p* < 0.001 and *p* = 0.001, respectively). The patients with severe pneumonia had higher rates of smoking and heart failure (*p* = 0.015 and *p* = 0.018, respectively). Handgrip strength (HGS) was found to be lower in the severe pneumonia group (*p* = 0.014).

**Table 2:** Comparisons of demographic, clinical, and comprehensive geriatric assessment characteristics between patients with mild and severe pneumonia

	Mild pneumonia (*n* = 36)	Severe pneumonia (*n* = 14)	*p*
Age, years	71.4 ± 6.0	74.8 ± 5.9	**0.041**
Sex, female	21 (58.3)	10 (71.4)	0.52
BMI (≥ 30 kg/m ^2^ )	13 (40.6)	3 (21.4)	0.32
Smoking	6 (18.8)	8 (57.1)	**0.015**
Polypharmacy (≥ 5 drugs)	17 (50.0)	10 (71.4)	0.17
Multimorbidity(≥ 2 chronic diseases)	29 (80.6)	11 (84.6)	0.75
Chronic obstructive pulmonary disease	9 (25.0)	7 (50.0)	0.089
Diabetes mellitus	23 (63.9)	8 (57.1)	0.66
Heart failure	1 (2.8)	4 (28.6)	**0.018**
Clinical frailty scale score	3.0 [1.0]	5.0 [2.0]	**0.002**
Living with frailty (CFS ≥ 4)	14 (42.4)	12 (85.7)	**0.006**
MNA-SF score	14.0 [1.0]	11.0 [3.0]	**< 0.001**
Malnutrition (MNA-SF ≤ 11)	3 (9.1)	8 (57.1)	**0.001**
Probable sarcopenia	12 (44.4)	9 (75.0)	0.096
Handgrip strength, kg	18.2 [10.3]	14.9 [11.4]	**0.014**
Chair stand test, sec	16.0 [5.8]	21.0 [20.1]	**0.031**
Albumin, g/dL	4.2 ± 0.9	4.2 ± 1.2	**0.003**
Hemoglobin, g/dL	13.6 [3.0]	11.7 [3.8]	**0.020**
Sedimentation rate, mm/h	12.0 [11.0]	25.0 [46.0]	**0.001**
Ferritin, ng/mL	19.0 [18.0]	125.0 [144.0]	**< 0.001**
C- reactive protein, mg/L	0.3 ± 0.6	1.7 ± 2.3	**0.001**

Variables are presented as n (%), mean ± SD, median [IQR] values and bold values indicate statistical significance (*p* < 0.05)

*MNA-SF* Mini-nutritional assessment short-form

In univariate/unadjusted regression analysis, heart failure (OR:14.0, 95% CI:1.40–39.81, *p* = 0.025), smoking (OR:5.06, 95% CI:1.24–20.63, *p* = 0.024), malnutrition (OR:13.3, 95% CI: 2.72–65.40, *p* = 0.001) and frailty (OR: 8.14, 95% CI: 1.57–42.33, *p* = 0.013) were found to be significant in increasing the severity of pneumonia. In the multivariate logistic regression adjusted for age, sex, and frailty status, malnutrition was independently associated with increased pneumonia severity (OR = 11.921, 95% CI: 1.592–89.285, *p* = 0.016). The CFS and MNA-SF scores were included in the model. Higher MNA-SF scores, indicating better nutritional status, were seen to be independently correlated with a lower risk of severe pneumonia (OR = 0.442, 95% CI: 0.242–0.806, *p* = 0.008). The results of the binary logistic regression analysis of factors associated with pneumonia severity are shown in Table 3.

**Table 3:** Binary logistic regression analysis of factors associated with pneumonia severity

	Odds ratio	95% CI	*p*
Model 1			
Age, years	1.120	0.975–1.287	0.11
Sex, female	0.784	0.141–4.354	0.78
Living with frailty	1.398	0.675–2.897	0.37
Malnutrition	11.921	1.592–89.285	**0.016**
Model 2			
Age, years	1.138	0.983–1.318	0.083
Sex, female	0.768	0.118–5.016	0.78
CFS, score	1.017	0.438–2.357	0.97
MNA-SF, score	0.442	0.242–0.806	**0.008**

Bold values indicate statistical significance (*p* < 0.05)

## Discussion

The main finding of this single-centre, retrospective study is that frailty and malnutrition impair the 1-year impact of PCV-13 vaccine in older adults. Several studies have shown that pneumococcal vaccines are effective in reducing the likelihood of bacterial pneumonia caused by S. pneumoniae (all Invasive Pneumococcal Disease (IPD), IPD caused by PCV13 serotypes.) and its associated complications, with reported efficacy of 60–80% in those aged ≥ 65 years and those with chronic diseases [25–28]. In the current study cohort, 50 of 407 older adults who had been vaccinated developed community-acquired pneumonia (CAP). Geriatric syndromes may influence the course and risk of pneumonia. Frailty has been shown to correlate with the CURB-65 score [29, 30], and to be significantly associated with mortality, hospital readmission rate, and length of hospital stay [31]. The current study findings indicate that frailty was more prevalent among patients with severe pneumonia, suggesting that frailty may affect the response to PCV13 in older adults.

Malnutrition is a leading cause of immunodeficiency worldwide and is thought to increase susceptibility to infection [32]. Malnutrition can adversely affect lung function by reducing ventilatory drive and respiratory muscle function, altering lung parenchyma, and suppressing lung defence mechanisms [33]. Malnutrition is known to cause immunodeficiency, and studies are needed to determine its effect on PCV13 vaccine response. A previous study found that children with malnutrition were hyporesponsive to PCV13 [34]. Although studies in the literature investigating the relationship between malnutrition and bacterial pneumonia have mainly been conducted on children, the current study results showed that malnutrition increases the severity of pneumonia, regardless of the patient’s age, sex, and frailty status. In addition, the severity of pneumonia increased as the MNA score decreased in this study. Some studies have also shown that malnutrition is associated with the development of pneumonia and an increase in adverse discharge status and long-term mortality risk in the geriatric population with CAP [35, 36].

Furthermore, another geriatric syndrome, sarcopenia (which manifests as a result of malnutrition), is also a risk factor for the development of pneumonia. A previous study reported that sarcopenia diagnosed by HGS was a risk for pneumonia [37, 38]. In the current study, HGS was found to be lower in the severe pneumonia group. Therefore, assessing and addressing sarcopenia may play a key role in reducing pneumonia severity among older adults, a relationship that should be further explored in future studies. The current study findings highlight that both malnutrition and frailty are strongly linked to pneumonia severity in older adults, underscoring their clinical relevance in hospitalization decisions. Malnutrition was seen to be independently associated with an increased risk of severe pneumonia, while higher MNA-SF scores, reflecting better nutritional status, were protective. Similarly, frailty was markedly more prevalent among those with severe pneumonia, aligning with evidence that frail older adults experience diminished physiological reserve and impaired immune responses, which exacerbate infection outcomes [39]. Previous studies have shown that frailty independently predicts poor prognosis, more extended hospital stays, and mortality in CAP [40, 41]. Likewise, malnutrition has been recognized as a modifiable risk factor that worsens infection outcomes and delays recovery. Therefore, integrating frailty and nutritional assessment into the routine evaluation of geriatric patients with pneumonia could help clinicians better identify those at higher risk for severe disease and guide early hospitalization or intensive monitoring strategies. Prevention, diagnosis, and treatment of malnutrition may be essential for improving outcomes in these patients, but further research is needed to investigate this relationship in older adults.

Previous studies have reported that breakthrough pneumonia is more common among individuals with multiple comorbidities, including chronic heart, lung, or liver disease, DM, alcoholism, and smoking, and that advanced age is an independent risk factor for hospitalization [26, 27, 42]. Consistent with previous research, the findings obtained in the current study indicate that comorbidities such as heart failure, DM, COPD, and smoking were associated with increased pneumonia severity and risk among older adults vaccinated with PCV-13. Therefore, older adults with chronic diseases should be considered a priority group for pneumonia prevention and post-vaccination follow-up.

With the plateauing of herd immunity achieved by PCV13, its limited immunogenicity against specific serotypes, and the continuing threat of infections caused by non-vaccine serotypes, new-generation conjugate pneumococcal vaccines such as PCV15 and PCV20 have been developed [43]. In 2021, the ACIP recommended PCV20 alone or PCV15 followed by PPSV23 for adults aged ≥ 65 years and for younger adults with underlying medical conditions or risk factors [7, 44]. PCV20, a 20-valent conjugate vaccine, expands coverage beyond PCV13 by including seven additional serotypes associated with adult disease, nearly doubling the proportion of vaccine-preventable invasive pneumococcal infections in older adults [45, 46]. Immunogenicity studies have demonstrated that PCV20 elicits strong opsonophagocytic antibody responses comparable to PCV13 for shared serotypes, while providing robust protection against additional ones [46]. Moreover, PCV20 has shown a safety and tolerability profile similar to that of PCV13 [10]. Owing to its broader serotype coverage and sustained immunogenicity, PCV20 offers enhanced protection for older adults and may reduce the need for subsequent PPSV23 boosters. In a review evaluating the immunogenicity and safety of PCV20 in adults aged ≥ 65 years, strong immune responses were detected in the first month of PCV 20 vaccination [11, 12]. Continued research into serotype-independent vaccine strategies remains essential to further decrease the global burden of pneumococcal disease.

This study had some limitations, primarily that PPSV23 and PCV20 vaccines were not available in Turkey at the time of the study, and therefore only older adults vaccinated with PCV13 were included in the study. PCV20 has only recently been introduced in Turkey. Another limitation of the study was the retrospective design and dependence on hospital records and telephone follow-up, which could have led to potential selection and recall biases. The lack of microbiological confirmation, including the absence of S. pneumoniae identification, may have limited pathogen-specific inferences and weakened the causal link to vaccine-related outcomes. Therefore, an overestimation in diagnosis may have occurred. However, the use of antibacterial treatment is highly restricted in Turkey, and treatment indications are determined by infectious disease specialists. Future studies incorporating microbiological confirmation are needed to better clarify this association. Selection bias may have occurred, as participants were recruited from those who presented at the geriatric medicine outpatient clinic, potentially representing a population with greater health concerns than the general older adult population. However, a strength of the study is that our geriatrics outpatient clinic is a referral centre, receiving patients from various regions of Anatolia, which may allow the study population to be representative of the broader older adult population in this region. Nevertheless, this study enrolled more than 400 consecutive individuals aged over 65 years who were followed up for up to 1 year after vaccination. To the best of our knowledge, this is the first study to have shown that breakthrough pneumonia diagnoses in PCV13 vaccine recipients were higher in frail and malnourished people. The fact that all those who were vaccinated underwent baseline CGA and were followed up for pneumonia outcomes for up to 1 year strengthens these findings.

In conclusion, the results of this study have shown that chronic diseases, especially DM, and COPD, are associated with a higher risk of pneumonia in adults aged > 65 years who have received the PCV-13 vaccination, and that the presence of malnutrition increases the severity of pneumonia, independent of age, sex and frailty. Severe pneumonia was observed to be more prevalent among patients living with frailty. The integration of geriatric principles into pneumonia prevention, including functional assessment, nutritional status and multidisciplinary care, is essential to improve outcomes in this vulnerable population. It is hoped that these findings will provide a comprehensive view of geriatric syndromes and contribute to the existing literature.

## Data Availability

Data are available from corresponding author upon reasonable request.

## Abbreviations

ADL: Activities of daily living
AMR: Antimicrobial resistance
CAP: Community–acquired pneumonia
CFS: Clinical frailty scale
CGA: Comprehensive geriatric assessment
COPD: Chronic obstructive pulmonary disease
CST: Chair stand test
CT: Computed tomography
DM: Diabetes mellitus
HGS: Handgrip strength
IPD: Invasive pneumococcal disease
MNA-SF: Mini–nutritional assessment short–form
PCV-13: Pneumococcal conjugate vaccine − 13
PPSV23: Pneumococcal polysaccharide vaccine − 23
S. pneumoniae: Streptococcus pneumoniae
SD: Standard deviation
SMMSE: Standardized mini–mental state examination
SPSS: Statistical package for social sciences
TUG: Timed–up–and–go test
YGDS: The yesavage geriatric depression scale
